# A New Methodology for Use by a Single Caregiver to Bathe Bedridden Elderly Persons Using Advanced Mechatronic Systems

**DOI:** 10.3390/healthcare7040124

**Published:** 2019-10-28

**Authors:** Karolina Bezerra, José Machado, Vítor Carvalho, Filomena Soares, Demétrio Matos, Marcelo Castro, Filipe Pereira, Hugo Lopes

**Affiliations:** 1 MEtRICs Research Center, School of Engineering, University of Minho, 4800-058 Guimarães, Portugal; karolceli@gmail.com (K.B.); filipe.as.pereira@gmail.com (F.P.); 2 Mechanical Engineering Department, School of Engineering, University of Minho, 4800-058 Guimarães, Portugal; marcelovieiracastro@gmail.com; 3 2Ai-EST-IPCA, Polytechnic Institute of Cávado and Ave, 4750-810 Barcelos, Portugal; vcarvalho@ipca.pt (V.C.); hugojrlopes@gmail.com (H.L.); 4 ALGORITMI Research Centre, School of Engineering, University of Minho, 4800-058 Guimarães, Portugal; fsoares@dei.uminho.pt; 5 Industrial Electronics Department, School of Engineering, University of Minho, 4800-058 Guimarães, Portugal; 6 ID+-ESD-IPCA, Polytechnic Institute of Cávado and Ave, 4750-810 Barcelos, Portugal; dmatos@ipca.pt

**Keywords:** caregiver, mechatronic assistive devices, bathing bedridden persons, methodology for bathing bedridden persons by a single caregiver

## Abstract

In the framework of this paper, we aimed to propose a methodology for giving baths to elderly, bedridden persons, when this task is performed by a single caregiver. Usually, two caregivers are required for nursing a bedridden patient, especially when certain important tasks are needed (e.g., bathing the patient), but this is not always possible. The entire study considers the primary user’s perspective—the caregiver—who is responsible for a wide range of tasks; thus, suffering physical and psychological exhaustion over time. A physical prototype has been developed for allowing caregivers to perform tests in a life-like environment, by means of the device and the methodology. This technology, therefore, will represent an important contribution to the quality of life of caregivers. Considering an increase in the share of the elderly population and the related problems that arise in daily care, this project intends to be beneficial contemporarily. The presented methodology has been successfully tested and validated.

## 1. Introduction

Currently the population aging trend in Europe shows that older persons (aged 65 or more) account for 19.2% of the total population (an increase by 0.3% over the previous year and by 2.4% over the former 10 years) [1].

It is considered that the long-term population age structure tends to demonstrate an increasing proportion of elderly persons, along with a decreasing proportion of active persons in the total population. It is estimated that the share of the population over 65 will increase in all EU Member States, European States and candidate countries [1,2].

In recent years, life expectancy has grown, and eventually, it has influenced the structural changes in families, which in turn end up resorting to support for care. In order to meet the needs inherent in social problems, there is a sustainable system with two support networks: the informal and the formal one [3]—which successively configure the types of caregivers [4,5,6].

Knowing that there are more and more persons who prefer to benefit from healthcare in their homes, this work was directed to the informal care network, covering the home environment, where the caregiver is the main source of direct care and psychological support [1,2,3,4,5,6]. Research has shown that there is an absence of methodologies related to the execution of activities needed to assist an elderly person in their everyday life, as in the bathing process [7,8,9,10,11,12,13]. In order to carry out the activities safely and ensure the health of the caregivers and of the bedridden, it was identified that the caregivers’ tasks require the aid of a technology-assisted methodology to allow for a better quality of life [7,8,9,10,11,12,13]. 

The circumstances in countries such as England, Australia, Canada, are favourable to socio-political strategies aimed at improving the last part of the life of bedridden persons and that encourage their treatment at home [14,15,16,17,18,19,20]. Several studies emphasize caregivers’ physical and emotional exhaustion and indicate the need to implement effective intervention strategies [21,22,23,24,25,26,27,28,29,30,31,32,33,34,35,36]. 

The informal caregiver is a single caregiver who is from the family. Therefore, it is necessary to elucidate the methodologies to assist informal caregivers in order to promote health policies and support them [14,15,16,17,18,19,20,37,38,39,40,41,42,43,44]. 

The opportunity to create the methodology is associated with the development of a mechatronic system for bathing with an embedded security function. The system consists of two parts: the water supply system and the bathing surface.

The development of such a system allows families and countries to reduce resources used for tending bedridden persons.

The remainder of the paper is organized as follows:

- Section 2 presents the hardware control system’s development;
- Section 3: New Methodology for Bathing—One Caregiver, describes the final methodology developed as a result of the overall studies;
- Section 4, Conclusion, summarizes the presented work and identifies directions for further improvements and developments.

## 2. Hardware Control System Development

This section consists of two parts—one dedicated to the water supply prototype and the second to the conceptual design of a new bath surface equipped with the functional skills that allowed for the development of the methodology associated with the bathing process and that proved the success of the design; in the final part is presented the step-by-step development of the aforementioned methodology.

### 2.1. Water Supply Prototype

**Figure 1:**
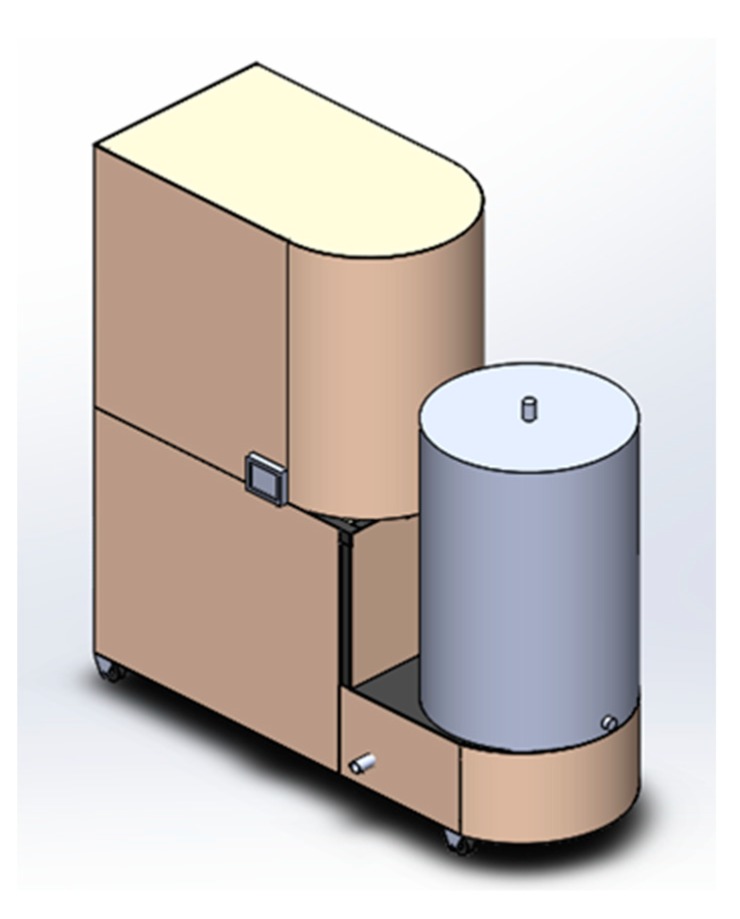
Water supply system concept.

The conceptual design of the proposed system is presented in Figure 1. The mechanical design of the water supply system, its requirements and specified parameters are detailed in [45].

The system has a robust structure due to the technical calculations that considered the need to accommodate a high quantity of water. Besides, it was necessary to choose from the components existing on the market, precisely to optimize its construction and to enable the first tests in a real environment. The material considered is Steel S 275 JR (1.0044 according to EN 10027-2: 2015).

**Figure 2:**
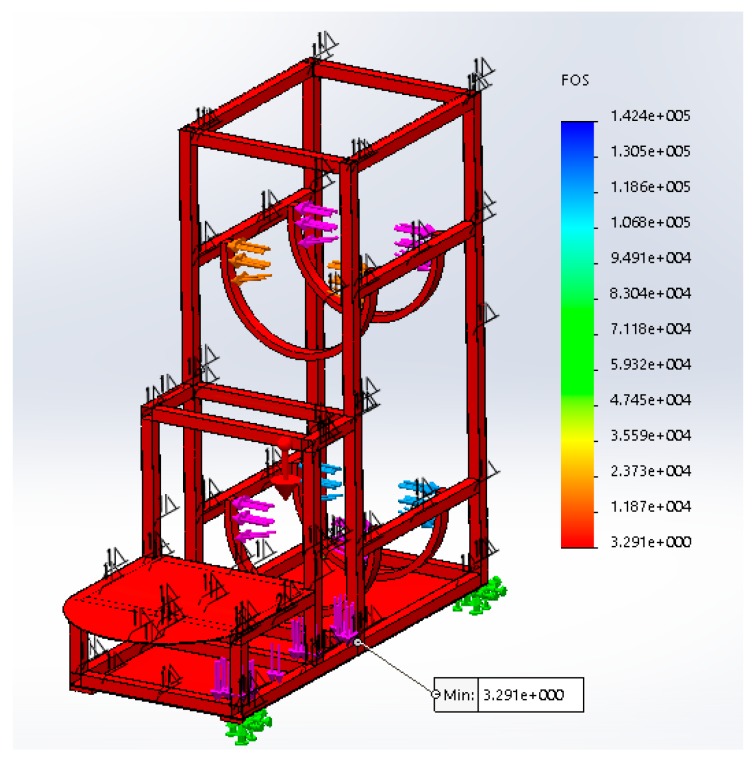
Result of the simulation of the pipe structure with a thickness of 0.5 mm.

SOLIDWORKS^®^ Simulation tool was used for static simulations, such as the analysis of the minimal thickness of the tube of the structure in mechanical terms (Figure 2). The forces used to test the structure were applied to the points of incidence with 1000 N because the safety coefficient is 3 and the total mass to be supported by the structure is estimated to be of 100 kg.

As a result of the simulation, the allowable tenure ratio and the yield stress of the structure were analysed with a result of the safety factor (SF) of 3.3 at the critical point indicated in Figure 2.

**Figure 3:**
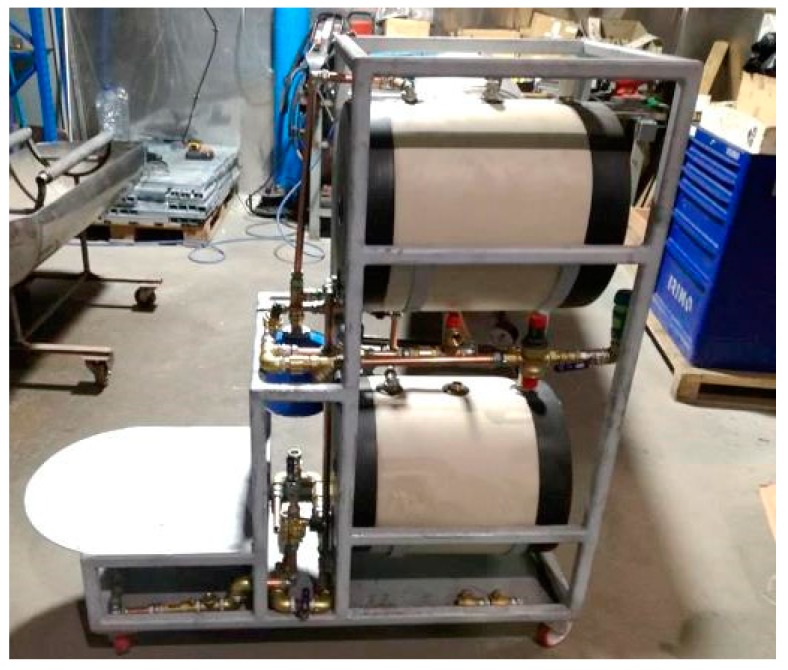
Mechanical system.

We opted for a galvanized welded tube structure to avoid corrosion, especially in the welded zones. Nevertheless, it has the advantage of facilitating the construction of the prototype, as welding is an easy manufacturing option, and in this case, the superior thickness of the structure facilitates the use of this manufacturing process. Therefore, as a function of the mechanical design guidelines, the final result of the mechanical system is presented in Figure 3.

**Figure 4:**
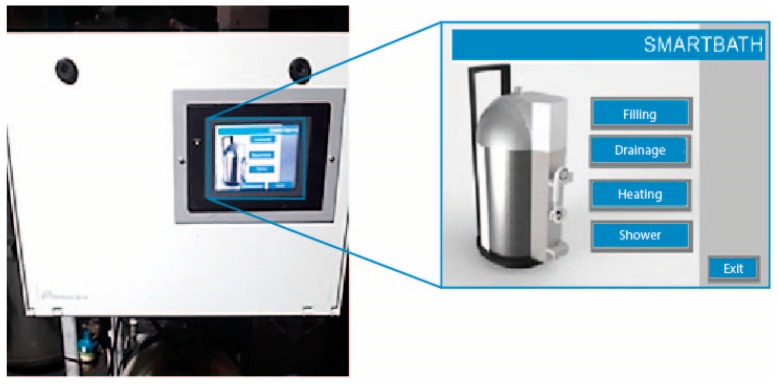
Electronic system and interface (controller).

The prototype uses an industrial programmable logic controller (PLC) and the controller program was specified using SFC [46] and it was implemented using Ladder programming language [47]. The HMI (human-machine interface) console was used, in order to allow the user to interact with the system developed and to display information. (Figure 4).

**Figure 5:**
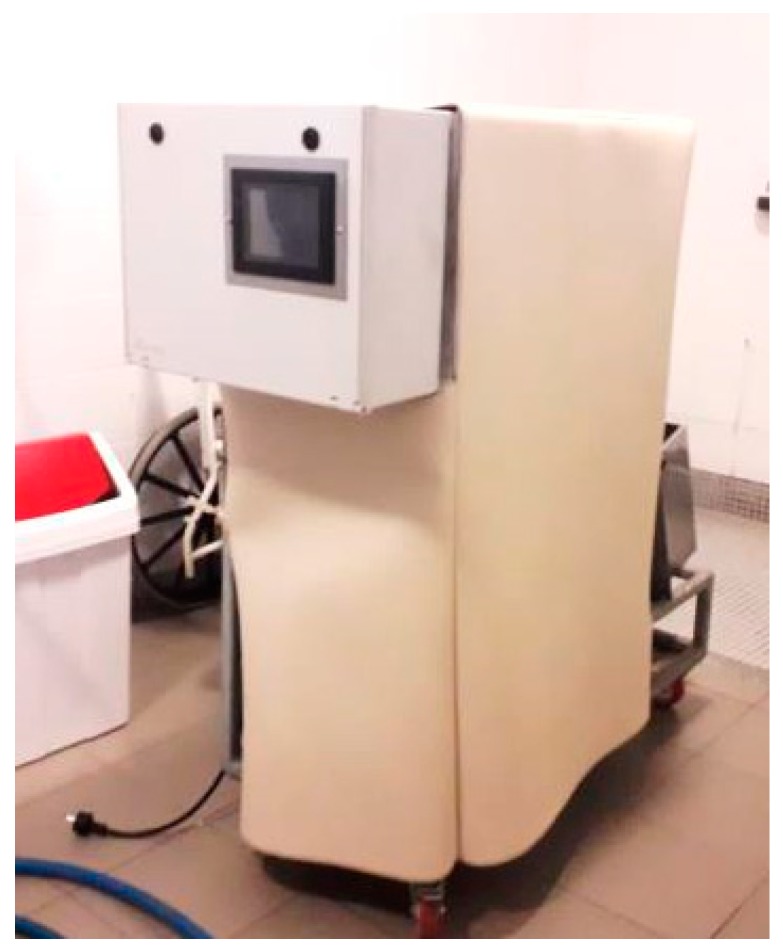
Prototype in test environment.

The final result of the prototype construction is shown in Figure 5.

The controller is responsible for assigning the desired functions; namely, filling the device with water, heating it, bathing and emptying the device. These steps are described as follows. First, the device must be connected to an external water supply. This connection is made via an adapter placed between the outlet of the water supply and the reservoir. In the next step, the device must be connected to the power supply; the caregiver activates the “Start” button placed on the control panel to boot the device. Then, he or she must select the “filling” function and the device starts the supply of water from the reservoir. When the sensors indicate that they are full, the user removes the connection between the device and the water supply. Then, the “heating” function must be selected, initiating heating of the water in the hot water tank. While the water is heating up, the caregiver can prepare the bedridden patient for the bath. The “bath” function is then activated, which requires setting the desired outlet temperature so that the system allows the bath to start. Finally, the last step is a manual process that requires the caregiver to perform the act of bathing the patient and control the outflow of water through the shower and the interface. The “emptying” function is activated when the bath finishes (the level sensors check if there is any water left in the tank); the remaining water must be evacuated. Afterward, finally, the water supply system is switched off.

### 2.2. Experimental Tests

The steps’ sequence runs until the tests are completed; the final results are described in the course of this section. The tests seek to establish a comparison between the expected result and the result obtained, in order to conduct a compliance analysis of the project parameters.

#### 2.2.1. Test of Technical Perception

Technical tests were carried out to guarantee the proper operation and to minimize the possible problems of this prototype. The technical test (Table 1) requires the evaluator to check the results obtained in the tests against the parameters set in the column of expected results and to evaluate whether there was compliance, or not, in terms of the operation of the prototype.

Based on the results of seven technical tests, it was concluded that the prototype meets most of the operating parameters, as can be verified in the column “compliance: yes or no.” It is important to consider that parameter 11 “output temperature set in controller” complies with the functionality, since it renders the final temperature of the resulting mixture of cold and hot water, which is of the utmost importance for patient safety. The “flow” parameter is also fulfilled because a maximum value of 6.5 L/min is obtained, and even lower values can be obtained if desired. However, it has been noted that there is still a need to improve the system so that the flow variations are smaller than those that took place during the tests. In the next prototype, greater attention should be given to parameters 7 “total mass of the device” and 10 “partial/total filling of the reservoirs.”

#### 2.2.2. The Caregiver’s Perception on the Usability of the System (Performed in an Institution)

The usability tests targeted caregivers of patients housed in a Portuguese institution in order to validate the use through the experience of the caregivers. In this phase, we obtained four results. The first observation relates to the formal aspect of the size of the equipment. Through the use of the prototype by the caregivers, the mobility parameters and the suitability of the tests in the real environment were validated. 

The test considered the functional aspects of the bathing experience, as assessed by the caregivers. They were asked to assess the aspects associated with this experienced via three answers corresponding to one of the two phases of the aspect. If an aspect was positive, they were asked to select “Yes.” If the aspect was not fully satisfactory, they were asked to select “More or Less,” and if it was negative, they had to select “No.” During the test procedure, all tests were satisfactory, with tests 2 and 4 performing better than tests 1 and 3. In the latter, there were certain features of the prototype that did not match the expected ones, but the “bathing” task was not compromised.

It proved the benefits of the fact that we could carry out the tests in the real environment: that made it possible to perceive the limitations and the positives aspects, and define the improvement directions for the evolution of the project.

### 2.3. Conceptual Design of a New Bathing Surface

Taking into account the limitations of bathing in any environment, the need to create a new concept was identified. Here, we describe the system design that gave rise to the concept of the bathing surface. Based on the requirements defined above, six concepts were developed (Table 2).

**Figure 6:**
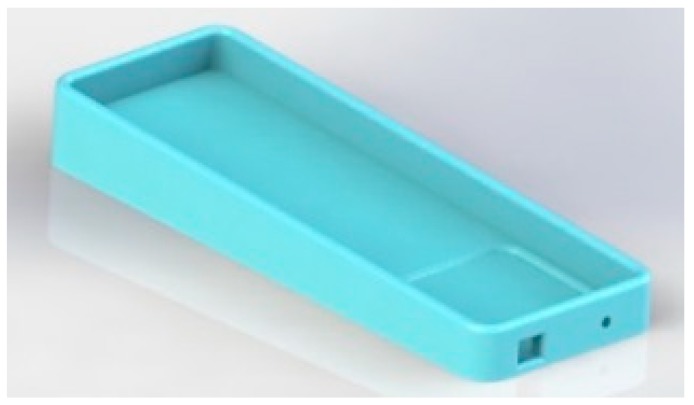
Surface concept 6.

Concept 6 is considered the conceptual surface that meets all requirements. It was considered for being inflatable and enabling mobility, hygiene and safety; moreover, it is the most suitable model for performing the bathing assistance function. The use of the surface corresponding to concept 6 is presented in Figure 6.

**Figure 7:**
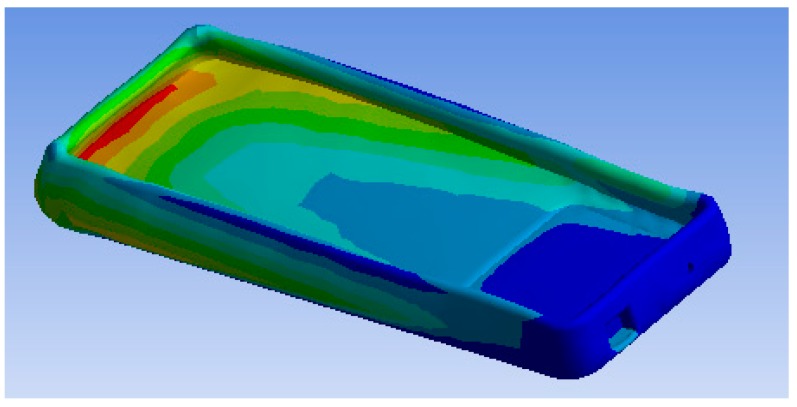
Ansys simulation result—deformation.

The pressure inside the inflatable area should be set at the time of use and it is related to the surface and the mass of the bedridden person [48]. The simulation of the use of this area by a person with the stipulated mass of 120 kg is presented in Figure 7. The isotropic and static (time invariant) distributed load was obtained through the finite element simulation in ANSYS software. It determined the linear static analysis to be the best approach to study the structure in conditions that simulate actual use. 

Evaluating the deformation and strain of a simulation required an inflatable structure between the two materials: the air that is inside the surface and the outside PVC material. Thus, the base was fixed and it was assigned a maximum load of 1177 N. In Figure 7 the largest deformation at the top, where there is a greater quantity of air, is identified, with a value of 0.0078 mm.

### 2.4. Cost of the Prototype Developed

Despite being the first version of the prototype, it is important to present an overview of its global costs. The authors would like to highlight that the optimization of costs and detailed value analysis has not been performed until this stage. The main goal of this prototype was the validation of the concepts presented in Table 3. The presented costs are an indication that the cost of final commercialized version will be much lower than this value. The next version of the prototype will include a rigorous value analysis and the global cost will be considerably lower.

The global cost of prototype developed (1st version)—jointly with the bathing surface—was 5814 Euros, according to the detailed description presented in Table 3.

The authors believe that it will be possible to considerably reduce the global cost of the final system, resulting in a lower final price of the version of the device that will be commercialized—about 3000 Euros (final consumer price). If this goal is achieved, it will be a great plus for families featuring bedridden persons, mainly because—with this solution—a single caregiver will be able to give a bath to a bedridden person while performing all the tasks needed. In addition, many families will have financial capacity to afford it.

From the point of view of society—and answering to a serious societal challenge: the high percentage of aged persons in developed countries—this device seems to be a very important solution to be used and will save funds in the social systems of those countries, since nowadays those persons are institutionalized, resulting in very high costs for the social systems of such countries.

### 2.5. First Studies and Tests

Complementarily to the system, a surface was proposed that could be mounted in any space, and could, that way, ensure a bathing system that could achieve a higher quality and that requires less effort on the part of the caregiver. This section describes a study that was carried out using a bathing surface already extant in the market [47], and thus, later, the development of the concept that best suited our needs. 

It is essential to take into account that in a case study, the value lies not only in studying a phenomenon, but also in its context. To be able to generalize the existence of a prior theory is important [48].

In this study, the EZ-BATHE^®^ surface [48] was used to validate the physical product of the surface in real environments in care institutions, via usability tests, performed by a single caregiver. Thus, based on the bed bath methodology, a sequence of tests was performed, following a methodology organized in five stages:

Stage 1: organization of the environment;

Stage 2: assembly;

Stage 3: use;

Stage 4: cleaning/drying;

Stage 5: disassembly.

**Figure 8:**
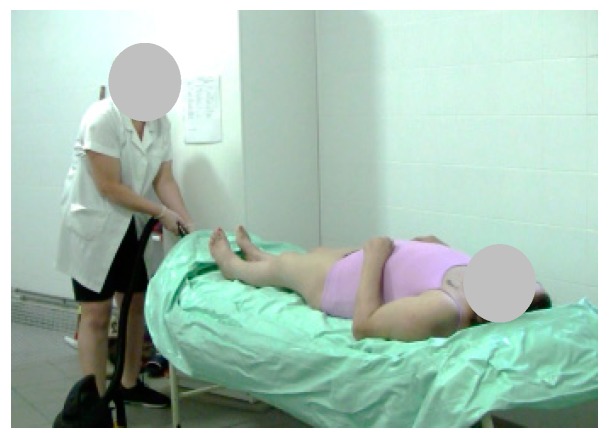
Step 1: Beginning of the filling process.

**Figure 9:**
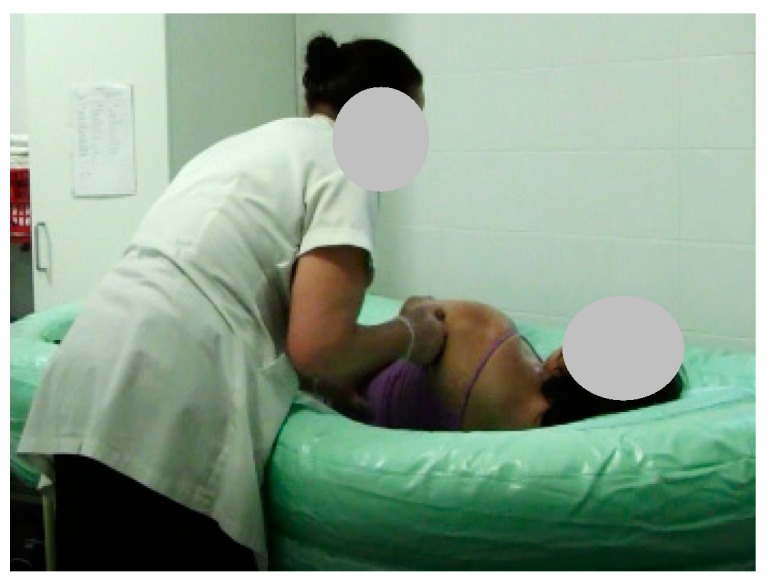
Step 5: The patient is washed on the sides and on the back.

**Figure 10:**
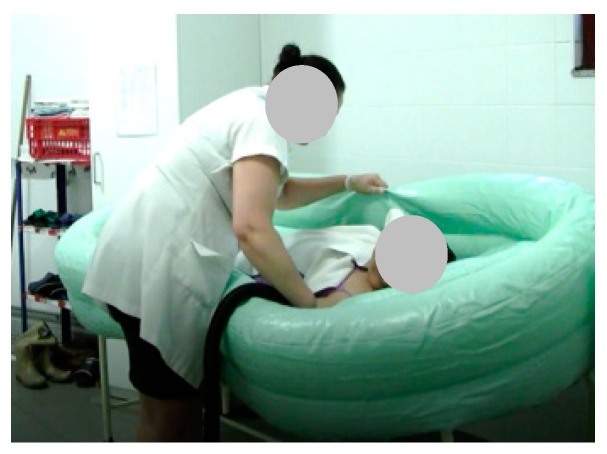
Step 13: The patient is dried.

**Figure 11:**
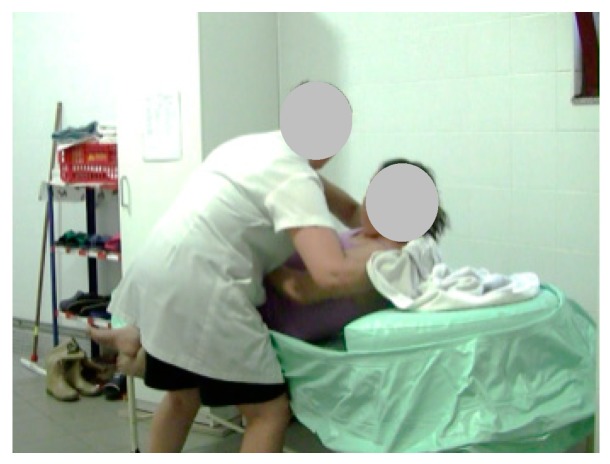
Step 21: The layer is removed.

As a result, we performed five tests; only three were performed by a single caregiver. As noted, during the test, the caregiver used as a base, a flat support structure. In the total of the five stages of the test 26 steps that were performed, the following should be noted: first, step 2 (Stage 2), the beginning of the filling process (Figure 8); then the emptying step 5 (Stage 3) was performed (Figure 9); to exemplify the drying of the bedridden, step 13 (Stage 4) is shown in Figure 10; and finally, stage 5 is exemplified with step 21, where the caregiver alone removed the layer from the surface (Figure 11). When the structure is being cleaned with the bedridden inside, the process is difficult and time consuming. The caregiver uses a leaning PVC structure, through which the remaining water can flow to a single location and the structure is then cleaned.

## 3. New Methodology for Bathing

The methodology development process was based on several studies through tests that featured the prototype and the bath surface in a real environment and performed by a single caregiver. It must be highlighted that this methodology was intended for use by a single caregiver.

### 3.1. Giving a Bath to a Patient via a Single Caregiver

This new methodology was developed to assist a single caregiver in a home environment, and it is designed to minimize the sequence of activities, reduce time and effectively eliminate physical difficulties. The advantage is that the use of this technology, coupled with manual labour, makes the caregiver more independent. 

Therefore, in this specific case, the overall system, the bath surface and the water supply system were integrated into the methodology in order to validate, in real environment, the different steps of the methodology that was developed, which was intended to be generic. To use the methodology, the following stages and respective tasks/steps must be performed:

#### 3.1.1. Stage 1: Assembling the Supply System

1. Connecting the device to a power source of 230 V–50 Hz;
2. Connecting to the hydraulic network of the bathroom with the aid of an adapter;
3. The interface indicates the filling of the tanks; (this procedure takes an average of 3 min, where the end of the procedure is automatic and signalled to the user by means of an audible alarm);
4. Disconnecting the power of the device 230 V–50 Hz;
5. Disconnecting the adapter from the hydraulic net, allowing the equipment to be transported to the place where the bathing process will take place.

#### 3.1.2. Stage 2: Using the Heating System

6. Connecting the device to a power source of 230 V–50 Hz;
7. Starting the operation of the water heating system (the process of heating the water will take approximately 45 min);
8. Checking the signal light (yellow); when it is on, the system has finished the heating process;
9. Starting the operation of the bathing system;
10. Choosing the optimal temperature for the bath, through the interface of the system;
11. Starting the bathing process manually by means of the shower.

#### 3.1.3. Stage 3: Assembly of Surface

12. Starting in parallel with task 6;
13. Organizing the environment with the following activities:
  - Taking off the bedclothes;
  - Changing the bedding;
14. In a process independent of steps 1 to 6, the surface must be mounted on a horizontal plane with dimensions of at least 500 × 1500 mm. It can be positioned directly beneath the user if he or she has a poor mobility.
15. The surface is inflated by means of an air pump with an electric drive compatible with the internal pressure of the surface;
16. Connecting the water-emptying surface to the lower side of the inflatable via a water outlet hose that connects the tank with the wastewater.

#### 3.1.4. Stage 4: Bath

17. Using the shower as described in task 11;
18. Washing the front of the body: the water is soaked with soap and then a circular movement is performed all over the body;
19. Washing parts of the sides (back) of the patient with the left hand resting on the right shoulder of the bed; the body of the bedridden person is pulled, and the lateral cleaning is performed. For the opposite side, mirrored operations apply;
20. Head cleaning: the caregiver should raise the head of the bedridden and use the shower to ensure the water supply; then, the caregiver puts on the shampoo; afterwards, he or she washes the head and uses the shower again to remove the water;
21. Washing the feet: the caregiver should use the shower, put the soap on, make circular movements, and use the shower again to clean up the soap.

#### 3.1.5. Stage 5: Surface Cleaning

22. Switch off the bath system via the controller interface;
23. After completion of the bathing process, the device should perform a complete drainage of surface water; (external or manual device);
24. The patient needs to be dried mechanically with a towel;
25. Drying the surface mechanically with a towel;
26. Switching off the drain system.

#### 3.1.6. Stage 6: Disassembly

27. Removing the surface under the bed;
28. Folding the surface, for storage;
29. Getting to the place where the emptying of water tanks can be performed;
30. Ending the shower procedure.

This methodology was designed to guide the caregiver, for him or her to be able to perform his /her bathing activities in any environment. It can be implemented in different environments, providing solutions to the problems of individuals and eliminating the physical limits of the caregivers themselves.

## 4. Conclusions

There are several alternative technologies used for tending bedridden patients, but there are no similar methodological alternatives. In this sense, this work consisted of several stages of development that began with the construction of the prototype of the water supply system that was proven to meet the idealized requirements; it is intuitive and ensures the needed functions of providing water and ensuring control of water temperature for the bathing process.

The execution of the prototype in a real environment allowed developing, improving and validating the proposed methodology. During tests, the caregivers were able to interact with the system in an easy manner. Moreover, one of the significant advantages of the prototype is that it is characterized by limiting the need for tasks and that it optimizes: the time needed to use the mechatronic system for ensuring water supply and water heating.

While performing the work—considering the use of the designed, built and tested equipment—it was possible to develop and validate a bathing methodology for bedridden persons. A new methodology was proposed to assist a single caregiver in performing the bedridden bathing activity in home settings. It is presented in brief through six basic stages:

- Stage 1: assembling the supply system;
- Stage 2: using the driver;
- Stage 3: surface mounting;
- Stage 4: performing the bathing;
- Stage 5: surface cleaning;
- Stage 6: disassembly.

It is assumed that caregivers with physical limitations will always look for the most practical way to perform the bathing activity, either manually or with the aid of a device. Therefore, simplifying the solutions and the level of effort will always make a difference in their activity.

Future work will consider the improvement of the project following the introduction of new functionalities in the prototype and further research into the bathing surface.

## Figures and Tables

**Table 1:** Final results of the technical test of the prototype.

Description of Parameters	Method	Expected Result	Obtained Result	Compliance (Yes or No)
Tank1-filling time	Level sensor	Temp1 ≤ 8 min	4 min 28 sec	Yes
Tank2-filling time	Level sensor	Temp2 ≤ 8 min	7 min 32 sec	Yes
Maximum temperature	Temperature sensor	T = 60 °C	60 °C	Yes
Ensuring a temperature under 42 °C after mixing	Temperature sensor	38 °C ≥ T ≤ 42 °C	35/37 °C	Yes
Output flow	Reservoirs	Value ≈ 5 L/min	≈6.5 L/min	Yes
Usage time	Timing	Temp ≥ 10 min	10–15 min	Yes
Total mass of the device	Balance	Max. 100 kg	≈150 Kg	No
Heat loss analysis: maximum 1 °C in 10 min	Temperature sensor	Temp ≈ 10 min	1 °C	Yes
Noise produced	Decibelimeter	60 dB (A)	47 dB (A)	Yes
Partial/total filling of reservoirs	Level sensor	Reading of level sensors	Does not indicate partial filling/Indicates total filling of reservoirs	No
Output temperature set in controller	Temperature sensor	Final temperature resulting from mixing the water, against the temperature set in the controller	Indicates the outlet temperature	Yes
Access to interior and all parts	User Perception	Assembly and disassembly	There is access to all parts	Yes

**Table 2:** Bathing Surface Concepts.

Number	Concept
1	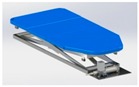
2	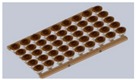
3	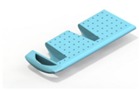
4	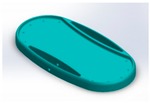
5	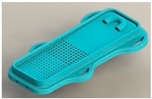
6	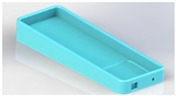

**Table 3:** Cost of prototype developed (1st version).

Description of Components	Cost (Euros)
Mechanical Structure	550
Mechanical components (pipes, accessories, etc.)	390
Programmable Controller	420
Electronic components (cards, temperature sensors, etc.)	768
Mechatronic components (filters, electro-valves, pumps, etc.)	1396
HMI	560
Tanks	260
Assembling work	1050
Bath Surface and accessories	420
**Total**	**5814**

## References

[1] World Health Organization (2002). A Contribution of the WHO to the Second United Nations World Assembly on Ageing. 2° United Nations World Assembly on Ageing.

[2] Eurostat (2009). Ambient Assisted Living Roadmap. https://ec.europa.eu/eurostat/statistics-explained/index.php?title=Population_structure_and_ageing/pt.

[3] World Health Organization (2015). World Report on Ageing and Health.

[4] INE (2011). Recenseamento da População e Habitação—Censos 2011-Índice de Dependência de Idosos (N. °). https://www.ine.pt/xportal/xmain?xpid=INE&xpgid=ine_indicadores&indOcorrCod=0006042&selTab=tab0.

[5] Christensen K., Doblhammer G., Rau R., Vaupel J.W. (2009). Ageing populations: The challenges ahead. Lancet.

[6] Pimentel L. (2001). O lugar do Idoso na Família: Contextos e Trajetórias.

[7] Silverstein M., Bengtson V.L., Putnam M., Putney N.M., Gans D. (2008). Handbook of Theories of Aging.

[8] Gillis K., Tency I., Roelant E., Laureys S., Devriendt H., Lips D. (2016). Skin hydration in nursing home residents using disposable bed baths. Geriatr. Nurs..

[9] Brito L. (2002). A Saúde Mental dos Prestadores de Cuidados a Familiares Idosos.

[10] Neno R. (2004). Male Carers: Myth or Reality. Nurs. Older Pers..

[11] Lage M.I. Cuidar dos cuidadores de idosos dependentes. Proceedings of the 5° Congresso Nacional de Psicologia da Saúde.

[12] Irene M., De Carvalho L.B. (2009). Entre os Cuidados e os Cuidadores: O Feminino na Configuração da Política de Cuidados às Pessoas Idosas.

[13] Silva TG Da Mocelin C., De Souza S.S., Madureira V.F., Celich KL S., Colliselli L. (2017). The care of dependent elderly on the family context. Rev. Pesqui. Cuid. Fundam. Online.

[14] Wang S.Z., Bolling K., Mao W.L., Reichstadt J., Jeste D., Kim H.C., Nebeker C. (2019). Technology to Support Aging in Place: Older Adults’ Perspectives. Healthcare.

[15] Waldow V.R., Luzatto S. (2001). O cuidado Humano: O Resgate Necessário.

[16] Brody E.M., Litvin S.J., Albert S.M., Hoffman C.J. (1994). Marital status of the daughters and patterns of pacient care. J. Gerontol. Soc. Sci..

[17] Cancion F.M., Stacey S.J.O. (1999). Caring and Gender (AltaMira P).

[18] da Miranda L.S.C. (2013). Os Cuidadores Informais na Prestação de Cuidados a Pessoas Idosas em Situação de Dependência Um estudo no Concelho do Montijo. MSc Thesis.

[19] Orso Z.R.A. (2008). Perfil Do Cuidador Informal De Idosos Dependentes Do Município De Veranópolis–Rs. MSc Thesis.

[20] de Carvalho J.M. (2013). Lévinas, Emmanuel. Humanismo do Outro Homem.

[21] de Ferreira M.L.S.N. (2014). A Vulnerabilidade do Cuidador Informal Como Foco dos Cuidados de Enfermagem. Ph.D. Thesis.

[22] Brummel-Smith K., Dangiolo M. (2009). Assistive Technologies in the Home. Clinics in Geriatric Medicine.

[23] Daniel K.M., Cason C.L., Ferrell S. (2009). Emerging Technologies to Enhance the Safety of Older Persons in Their Homes. Geriatr. Nurs..

[24] Nelson A., Powell-Cope G., Gavin-Dreschnack D., Quigley P., Bulat T., Baptiste A.S., Friedman Y. (2004). Technology to promote safe mobility in the elderly. Nurs. Clin. N. Am..

[25] Healthcare Commission State of Healthcare. 2008. London. https://assets.publishing.service.gov.uk/government/uploads/system/uploads/attachment_data/file/248381/0011.pdf.

[26] Department of Health (2000). The NHS Plan: A Plan for Investment, a Plan for Reform. BMJ.

[27] Baker M. (1999). Argumentation and Constructive Interaction. Studies in Writing. Foundations of Argumentative Text Processing.

[28] Pegram A., Bloomfield J., Jones A. (2007). Clinical skills: Bed bathing and personal hygiene needs of patients. Br. J. Nurs..

[29] Philip P. (2013). Department of Health’s Annual Report 2012/13. Health’s Annual Report. http://www.health.vic.gov.au/about_us/annual_report.

[30] Baker F., Smith L., Stead L. (1999). Giving a blanket bath--2. Nurs. Times.

[31] de Leon C.M. (2005). Social engagement and successful aging. Eur. J. Ageing.

[32] Cho J., Martin P., Poon L.W. (2015). Successful aging and subjective well-being among oldest-old adults. Gerontologist.

[33] Westfall J.M., Mold J., Fagnan L. (2007). Practice-based research—“Blue highways” on the NIH roadmap. J. Am. Med Assoc..

[34] Active Standard ASTM F2761-09(2013) (2009). Medical Devices and Medical Systems—Essential Safety Requirements for Equipment Comprising the Patient-Centric Integrated Clinical Environment (ICE)—Part 1: General Requirements and Conceptual Model. https://www.astm.org/Standards/F2761.htm.

[35] Salman O., Carvalho V., Bezerra K., Soares F., Machado J., Leão C.P. (2015). Design of a conceptual bed mattress for reducing pressure on bony prominences. ASME Int. Mech. Eng. Congr. Expo. Proc. (IMECE).

[36] Kärki S., Lekkala J. (2006). Pressure mapping system for physiological measurements. Imeko World Congr. Metrol. Sustain. Dev..

[37] Whitehead P.J., James M., Belshaw S., Dawson T., Day M.R., Walker M.F. (2016). Bathing adaptations in the homes of older adults (BATH-OUT): Protocol for a feasibility randomised controlled trial (RCT). BMJ Open.

[38] Botia J.A., Villa A., Palma J. (2012). Expert Systems with Applications Ambient Assisted Living system for in-home monitoring of healthy independent elders. Expert Syst. Appl..

[39] Golding-Day M., Whitehead P., Radford K., Walker M. (2017). Interventions to reduce dependency in bathing in community dwelling older adults: A systematic review. Syst. Rev..

[40] Lieberman J.A., Stuart M.R., Lieberman J.A. (1999). The BATHE Method: Incorporating Counseling and Psychotherapy into the Everyday Management of Patients. Prim. Care Companion J. Clin. Psychiatry.

[41] Anacker H., Dumitrescu R., Gausemeier J., Iwanek P., Schierbaum T. (2014). Methodology for the identification of potentials for the integration of self-optimization in mechatronic systems. Procedia Technol..

[42] Haan B.D., Researcher P. A Simple Classification Approach to Build a Bathtub. Proceedings of the 2008 Annual Reliability and Maintainability Symposium, RAMS ’08.

[43] Dilma P.D.C. (2012). Avaliação Inicial Do Familiar Cuidador: Estudo De Adequação De Um Instrumento. Escola Superior de Enfermangem do Porto. https://comum.rcaap.pt/bitstream/10400.26/9341/1/Tese_mestrado_trabalho_Finalissimo%5B1%5D.pdf.

[44] Ministério da Saúde (2008). Guia prático do cuidador/Ministério da Saúde, Secretaria de Atenção à Saúde. Secretaria de Gestão do Trabalho e da Educação na Saúde.

[45] Svensson M.P.-Å. (1999). IEC 60848—Specification Language GRAFCET for Sequential Function Charts.

[46] Antonsen M.T. (2018). PLC Controls with Structured Text (ST).

[47] Z-BATHE®, (N.D.). https://www.ezaccess.com.

[48] Bezerra K., Machado J., Carvalho V., Castro M., Costa P., Matos D., Soares F. (2017). Bath-ambience—A mechatronic system for assisting the caregivers of bedridden persons. Sensors.

